# Autologous minced cartilage implantation in osteochondral lesions of the talus—does fibrin make the difference?

**DOI:** 10.1007/s00402-025-05762-7

**Published:** 2025-01-25

**Authors:** Jan Kühle, Ferdinand C. Wagner, Samuel Beck, Lukas Klein, Lisa Bode, Kaywan Izadpanah, Hagen Schmal, Nils Mühlenfeld

**Affiliations:** 1 University Medical Center Freiburg, Freiburg, Germany; 2 Odense University Hospital, Odense, Denmark

**Keywords:** Ankle surgery, Osteochondral lesion, Cartilage repair, Minced cartilage

## Abstract

**Introduction:**

Implantation of minced cartilage is a one-step-procedure that leads to satisfactory results in osteochondral defects.

**Material and methods:**

A retrospective review was performed on a consecutive cohort of patients that received minced cartilage with fibrin (MCF), minced cartilage with membrane and fibrin (MCMF) and minced cartilage with the “AutoCart”-procedure (MCAC) between January 2019 and December 2023. Radiological outcome parameters were evaluated via Magnet-Resonance-Tomography (MRI) within one year using Ankle-Osteoarthritis-Scoring-System (AOSS).

**Results:**

Final data included 25 patients: 13 women and 12 men with a mean age of 28.2 years ± 10.9(range:18–51). Mean defect size was 103.6mm^2^ ± 59.0(95% CI = 79.2–128.0), mean depth 5.2 mm ± 3.6(3.7–6.6). Differences between groups in defect sizes were not significant (p = 0.999). MCF was carried out in 9(36.0%), MCMF in 7(28.0%) and MCAC in 9(36.0%) patients. Mean postoperative AOSS values were 2.6 ± 1.9(95%CI:1.1–4.1) for MCF-patients, 3.3 ± 2.9(0.6–5.9) for MCMF patients and 3.9 ± 2.8(1.7–6.1) for MCAC-patients, respectively. Differences were not significant (p = 0.639). Revision surgery because of symptomatic non-integration of the graft or ventral impingement occurred in 5(20%) of cases – all part of MCAC-patients that did not receive additional fibrin fixation as the top layer of transplant (p < 0.05).

**Conclusions:**

Autologous minced cartilage implantation seems to be sufficient as a viable, one-step treatment for osteochondral lesions of the talus. It leads to low complication rates and excellent AOSS values within a 12-month period whereas the application of fibrin as the last fixation layer seems to be advisory, which demands further investigation.

**Supplementary Information:**

The online version contains supplementary material available at 10.1007/s00402-025-05762-7.

## Introduction

The implantation of autologous chondrocytes is known to be an excellent cartilage reconstruction technique to prevent premature osteoarthritis [1, 2]. The approach, using the application of minced cartilage is a simple as well as cost effective one-step procedure that leads to satisfactory results, even in larger osteochondral defects [3]. To date, results have mainly been demonstrated following knee joint pathologies, still lacking in long term outcomes [3–6]. It provides an excellent and cost-efficient alternative to other resource- and time-intense multi-step procedures [1, 7–9] and shows excellent biological potential as chondrocytes establish a de-novo extracellular matrix via outgrowth, proliferation, and differentiation [6, 10, 11]. For fixation of the cartilage fragments different techniques have been proposed, such as fibrin glue alone or in combination with a membrane [1, 4]. Another well-established method for the treatment of osteochondral defects in the knee is the AutoCart^™^-Procedure by Arthrex (Naples, Florida, USA). Hereby, the cartilage damage is treated exclusively with the patient's own material. Small cartilage particles are collected, mixed with platelet rich plasma (PRP) and reapplied to the lesion area. Their fixation is performed with autologous thrombin solution as well as an additional layer of PRP/thrombin mixture [12]. This has been reported to be low-cost and effective, however, lager study data are still not available, especially not for the ankle-joint [13].

Overall, there is only little data available to support the use of minced cartilage as a reliable surgical treatment method in osteochondral defects of the ankle and especially the talus. The fixation methods using fibrin only or in combination with a membrane have not yet been compared scientifically or reported on a larger case series. Consequently, there is need for more data evaluating the outcomes of this relatively new procedure as a treatment of osteochondral defects in the talus. This may be of clinical relevance to facilitate surgical decision-making.

Hence, the primary objective of this study was to analyze the consolidation rates of osteochondral defects following treatment with minced cartilage. This was evaluated via magnet resonance imaging (MRI) in a consecutive series of 25 patients. Differences in MRI-outcome (primary outcome) and the surgical revision rate (secondary outcome) within one year after surgery between the following treatment procedures were compared and evaluated:

- Minced cartilage with fibrin (MCF),
- Minced cartilage with membrane and fibrin (MCMF) and
- Minced cartilage with the “AutoCart^™^” procedure (MCAC).

## Methods

This study followed the STROBE guidelines for observational studies (Strengthening the Reporting of Observational Studies in Epidemiology) and the RECORD guidelines (Reporting of studies Conducted using Observational Routinely collected Data) [14, 15].

A retrospective review was performed on a consecutive cohort of all patients that received surgical treatment of osteochondral defect in the talus with Minced cartilage with fibrin (MCF), minced cartilage with membrane and fibrin (MCMF) as well as minced cartilage with the “AutoCart^™^” procedure (MCAC) at the authors’ institution between January 2019 and December 2023. Patients were identified via a retrospective query of the hospital electronic medical records using the International Classification of Diseases Version 10 (ICD-10) codes of the German Diagnosis Related Groups (G-DRG). Patients’ characteristics, disease-specific information, radiologic characteristics, type of surgical management and outcomes were abstracted and transferred to an electronic spreadsheet. Patients ≥ 18 years of age were eligible for inclusion. Exclusion criteria were age < 18 years, tumor disease, ubiquitous talus necrosis, corticosteroid therapy, diabetes and loss of follow-up.

Patients underwent standard radiological diagnosis including x-rays in two planes and MRI preoperatively and within 6–12 months after surgery. All MRI data (coronary and sagittal T1- and T2-weighted sequences) were evaluated by a specialized radiologist and a specialist foot and ankle surgeon independently. With the data from MRI, defect sizes were measured and the Ankle Osteoarthritis Scoring System (AOSS)—scores (primary outcome) were calculated as described by Schmal et al. [16, 17]:

1. Depth of cartilage damage: grade 0 (0 points): no, grade 1 (1 point): < 50% of total cartilage depth, grade 2 (2 points): > 50%, grade 3 (3 points): full thickness cartilage defects. The depth of cartilage loss was qualitatively rated in relation to the height of the adjacent intact cartilage or the expected, normal cartilage contour. In doubt, the sagittal T2-weighted sequence was used for the final decision.
2. Defect of the subchondral bone: grade 0 (0 points): no, grade 1 (1 point): minimal (< 2 mm), grade 2 (2 points): moderate (2–5 mm), grade 3 (3 points): severe (> 5 mm). The depth of the osseous component of the osteochondral defect was scored by estimating the distance between the actual osteochondral defect and the extrapolated subchondral cortex, mainly based on evaluation of the coronary or sagittal T1-weighted sequences.
3. Osteophytes: grade 0 (0 points): no, grade 1 (1 point): minimal (< 3 mm), grade 2 (2 points): moderate (3–5 mm), grade 3 (3 points): severe (> 5 mm). Size was measured from the base to the tip of the osteophyte; baseline was defined as the natural course of the bone.
4. Subchondral cysts (largest diameter): grade 0 (0 points): no, grade 1 (1 point): minimal (< 3 mm), grade 2 (2 points): moderate (3–5 mm), grade 3 (3 points): severe (> 5 mm). Subchondral cysts were defined as structures of high signal intensity on T2-weighted images in the cancellous bone underlying the joint cartilage.
5. Bone marrow edema (largest diameter): grade 0 (0 points): no, grade 1 (1 point): minimal (< 5 mm), grade 2 (2 points): moderate (5–20 mm), grade 3 (3 points): severe (> 20 mm). Bone marrow edema was assessed as an area of increased signal intensity on T2-weighted images in the subchondral cancellous bone.
6. Anterolateral or anteromedial meniscoid: 0 points: no, 1 point: yes. MR images were assessed for appearance of pathological anterolateral or anteromedial soft tissue structures.
7. Effusion: 0 points: no, 1 point: yes. If more than a small, physiological sliver of synovial fluid was observed in the T2 images, joint effusion was assumed to be present.
8. Loose joint bodies: 0 points: no, 1 point: yes.
9. Synovitis: 0 points: no, 1 point: yes. Synovitis was evaluated on sagittal images and was reflected by thickening and irregularity of the normally pencil-thin rim of high signal intensity synovium.
10. Soft tissue cysts (Baker cyst analog): 0 points: no, 1 point: yes. These structures may be considered as excrescences originating from the joint capsule. They are depicted as a circumscribed mass with intermediate signal intensity on proton density-weighted and high signal intensity on T2-weighted sequences and are usually observed in the triangle of calcaneus and Achilles tendon.

Evaluation was performed by three different specialized orthopedic ankle surgeons. Observers were masked to the patients' biometrical and descriptive data.

Secondary outcome was surgical revision rate, which occurred within the observation period after initially receiving the minced cartilage procedure.

### Surgical procedure and rehabilitation protocol

All patients were treated by 4 different specialized orthopedic foot and ankle surgeons. In larger defects where an insufficient bone stock was present, cancellous bone was additionally used as a base layer for the chondrocyte refixation. Cancellous bone was harvested from the tibial head, the distal tibia, or the iliac crest. In patients, that presented with an osteochondral defect of the medial talus that could not be reached through the ventral capsule access, an osteotomy of the medial malleolus was performed, which was refixed with two lag screws afterwards. Before transplantation of cancellous bone and minced cartilage, broken subchondral bone was either partially resected to freshen the receiver site or K-wire drill holes (1.6 mm with 3–4 mm spacing) were installed in cases where the bone surface was still intact.

#### Minced cartilage with fibrin (MCF)

**Fig. 1:**
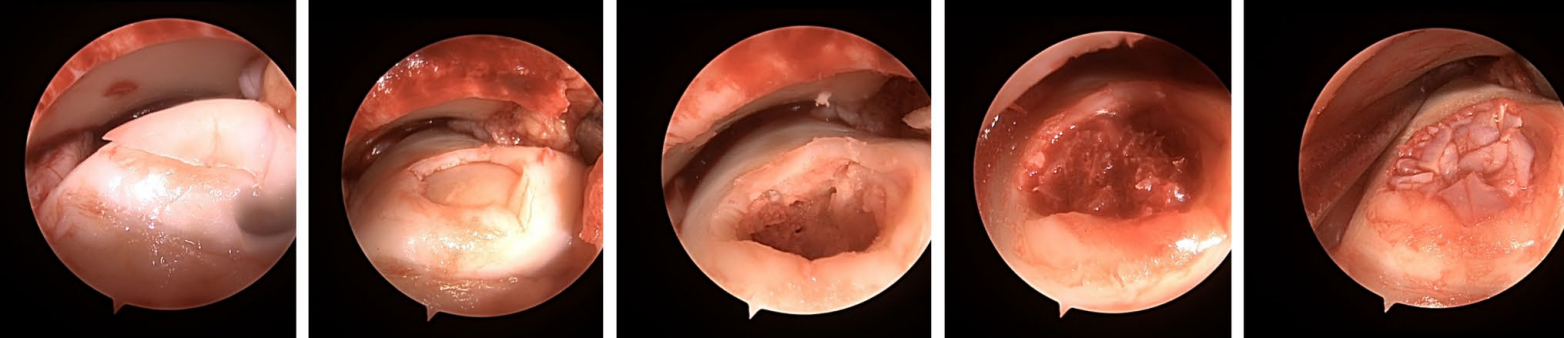
Intraoperative arthroscopic pictures of the preparation and minced cartilage implantation in a patient with an osteochondral lesion of the medial talus size 15 × 15 × 8 mm. Fixation was done with fibrin

With a shaver or a knife, cartilage particles from the affected area were resected, collected, and then retransferred to the area after implantation of cancellous bone if necessary. They were then refixated with fibrin to form a stable surface for chondrocyte regrowth and differentiation (Fig. 1).

#### Minced cartilage with membrane and fibrin (MCMF)

This procedure is comparable to MCF, but the cartilage particles were additionally refixated with a collagen matrix membrane (Chondro-Gide, Geistlich Pharma, Wolhusen, Switzerland) as well as fibrin to form a stable matrix-chondrocyte-conglomerate for cartilage regrowth.

#### *Minced cartilage with the “AutoCart*.^*™*^*” procedure (MCAC)*

Using a shaver, 3- or 4- millimeter particles of cartilage were resected, collected, and then transferred to a syringe for application. After mixing with PRP, particles were transferred to the lesion area and then fixated with an autologous thrombin solution produced by the “Thrombinator^™^” system. Finally, everything was sealed with an additional layer of PRP/thrombin mixture[12]. In four patients, an additional fibrin layer was added.

All additional pathologies such as ligament instability or axis deviations were treated when necessary. After surgery, patients’ feet and ankles were immobilized with a strong ankle boot for 6 weeks with mobilization out of the boot without mobility restriction after two weeks. Mobility training out of the cast was allowed right away after surgery to prevent stiffening. Patients were instructed to avoid any strong axial impact loads for 6 months.

### Statistical analysis

Since the examined surgical procedure is novel and no other data were available for comparison, all patients available were included and no power analysis was carried out.

All variables were evaluated for distribution of normality using a combination of histograms, quantile–quantile (Q–Q) plots and Shapiro–Wilk tests. Descriptive statistics were summarized as means and standard deviations for quantitative variables and as counts and frequencies for categorical variables across all variables. All Data followed normal distribution. Because of the small sample size, male and female data were combined to one set of data. Differences between MRI data in the impact groups were compared using Kruskall-Wallis-Test. Incidences were compared using a R × C table with a chi square test. Simple logistic regression analysis was performed to evaluate the relationship between incidences and patient age, -sex, as well as the defect size. A Spearman’s correlation was conducted to evaluate the relationship between defect size and AOSS, as well as the relationship between defect depth and AOSS. Tests were calculated two-tailed using a 95% confidence level. P-values < 0.05 were considered significant. Statistical analyses were performed with Graphpad Prism 10 (Graphpad, CA, San Diego).

### Compliance with ethical standards

Approval from the institutional review board was obtained before performing this retrospective study (24–1072-S1). The study was performed in accordance with the ethical standards of the institutional and national research committee and with the 1964 Helsinki declaration and its later amendments. This research did not receive any specific grant from funding.

Informed consent was not obtained as a study was done in an anonymized retrospective manner.

## Results

### Sociodemographic data and defect location

**Fig. 2:**
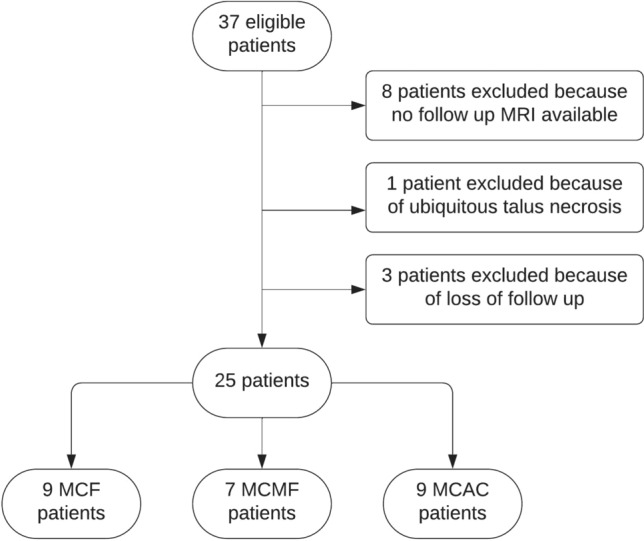
Patient flow chart demonstrating eligibility process for inclusion of data sets in this study

After the retrospective query of the hospital electronic medical records data included 37 patients of which 8 were excluded from the data set as they did not yet receive their 12-month follow-up MRI after surgical treatment. One patient received minced cartilage procedure as a rescue therapy because of ubiquitous talus necrosis and was excluded. Three patients were additionally excluded as they did not reappear to clinical or radiological follow-up. Final data included 25 patients: 13 women (52.0%) and 12 men (48.0%) with an osteochondral defect of the talus that received surgical treatment with one of the minced cartilage procedures (Fig. 2). Mean age of the included patients was 28.2 years ± 10.9 (range: 18–51). In 80.0% (n = 20) of the patients, the osteochondral defect was documented in the medial part of the talus, in 12.0% (n = 3) in the lateral and in 4.0% (n = 1) in the central part respectively. One patient had an osteochondral defect in the lateral and the medial shoulder of the talus (4.0%).

### Treatment procedure and defect size

Minced cartilage with fibrin-fixation (MCF) was the carried out in 36.0% (n = 9) of the cases, while minced cartilage with the use of a membrane and fibrin-fixation (MCMF) was performed in 28.0% (n = 7) and minced cartilage combined with the “AutoCart^™^” procedure (MCAC) in 36.0% (n = 9) respectively. Cancellous bone was harvested in 92.0% (n = 23) of the patients to build up the correct height of the articular surface of the talus. Bone was harvested from the proximal tibia in 64.0% (n = 16), the distal tibia in 20.0% (n = 5) and the iliac crest in 8.0% (n = 2) of cases.

Mean defect size measured preoperatively via MRI was 103.6 mm^2^ ± 59.0 (95% CI = 79.2–128.0) with a mean depth of 5.2 mm ± 3.6 (3.7–6.6) for the whole cohort. In MCF patients, mean defect size was 103.8 mm^2^ ± 55.3 (61.3–146.3) with an average depth of 4.6 mm ± 1.9 (3.1–6.1), in MCMF patients 104.4 mm^2^ ± 53.8 (54.7–154.2); 4.7 mm ± 1.4 (3.4–6.0) and in MCAC patients 102.8 mm^2^ ± 72.3 (47.2–158.3); 6.1 mm ± 5.6 (1.8–10.4) respectively. Differences between patient groups in defect sizes were not significant (p = 0.999).

Minimal invasive calcaneus-osteotomy to correct axis deviations were performed in three patients—one in each of the patient groups. Additionally, two lateral and one medial open ligament reconstruction were performed: one in the MCMF group and two in the MCAC group (Table 1).

**Table 1:** Number and percentages of concomitant surgeries for each group

Group	Minimal invasive calcaneus osteotomy n (%)	Lateral ligament reconstruction n (%)	Medial ligament reconstruction n (%)
MCF	1 (11.1)		
MCMF	1 (14.3)	1 (14.3)	
MCAC	1 (11.1)	1 (11.1)	1 (11.1)

### AOSS score derived from MRI

**Fig. 3:**
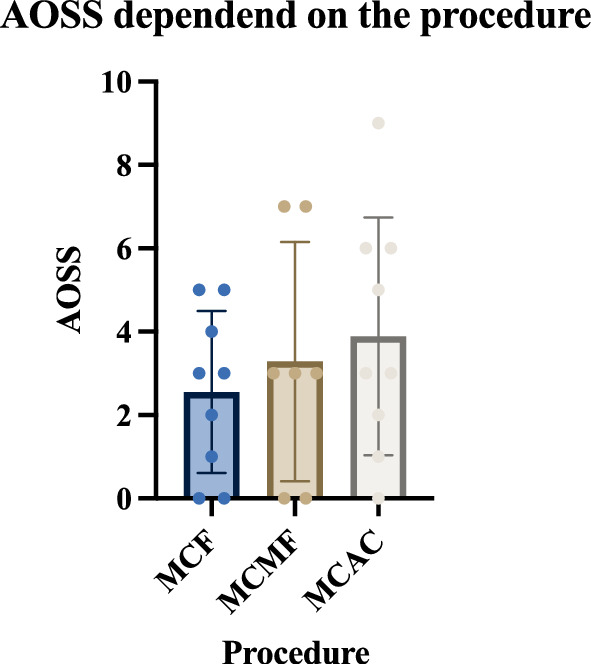
Scatter blot with bar of the values of Ankle Osteoarthritis Scoring System (AOSS) for each of the patients with groups mean and 95% Confidence Intervals, depended on the fixation procedure. Mean Score was 3.2 ± 2.5 (95% CI: 2.2–4.3) for the whole cohort. Differences between means were not significant (p = 0.639)

Patients reported for follow-up MRI at 10.7 ± 2.9 months postoperatively (range 6–13 months). The radiological outcome data derived from postoperative MRI are presented in Fig. 3. Mean AOSS for the whole cohort was 3.2 ± 2.5 (95% CI: 2.2–4 0.3). MCF patients presented with a mean score of 2.6 ± 1.9 (1.1–4.1), MCMF patients with 3.3 ± 2.9 (0.6–5.9) and MCAC patients with 3.9 ± 2.8 (1.7–6.1) respectively. The difference in AOSS between groups were not significant (p = 0.6392).

A Spearman’s correlation was conducted to evaluate the relationship between defect size and AOSS, as well as the relationship between defect depth and AOSS. The relationship between defect size and AOSS was not significant: rs =− 0.3021; p = 0.142. There was no significant relationship noted between defect depth and AOSS: rs = 0.05538; p = 0.793.

### Complications

Overall, 5 patients (20.0%) were not satisfied with the outcome of the procedure and underwent revision surgery. All these patients (100.0%) received minced cartilage transplantation with AutoCart^™^ procedure (MCAC Patients). Further analysis revealed that these 5 patients did not receive fibrin fixation as the last layer of graft fixation (p < 0.05). In MCAC patients that received additional fibrin fixation, there was no complication observed. Three patients with complications presented with insufficient graft-integration and progressive osteochondrosis and had to undergo revision of the minced cartilage. Two patients suffered from ventral impingement pain as well as stiffening of the ankle because of an osteophyte and underwent surgical osteophyte resection along with arthrolysis. Complication rate was not correlated to patient age (p = 0.105), sex (p = 0.138) or defect size (p = 0.054). All wounds healed primarily without wound healing disorders (Table 2).

**Table 2:** Demographic data, defect location, defect size and depth, as well as fibrin fixation, AOSS scores and Complication of the whole cohort for each patient

Group	No	Age (y)	Sex	defect	size (mm^2^)	depth (mm)	Fibrin	AOSS	Complication
MCF	1	31	m	med	225	8	yes	4	
	2	18	f	med	120	6	yes	0	
	3	21	f	med	96	3	yes	0	
	4	18	f	lat	96	5	yes	2	
	5	38	m	med	36	2	yes	5	
	6	19	f	med	117	5	yes	1	
	7	18	f	med	108	5	yes	3	
	8	50	m	med	36	5	yes	3	
	9	23	m	lat	100	2	yes	5	
MCMF	10	18	f	med	60	5	yes	7	
	11	18	f	med	96	4	yes	0	
	12	22	f	med	65	6	yes	3	
	13	31	m	med	102	5	yes	7	
	14	26	m	med	152	5	yes	0	
	15	31	f	med	200	6	yes	3	
	16	27	f	med	56	2	yes	3	
MCAC	17	26	f	med	200	8	yes	0	
	18	25	m	med	50	20	**no**	9	regenerate insufficiancy
	19	51	f	med	32	2	**no**	5	ventral impingement
	20	31	m	lat	32	3	**no**	3	regenerate insufficiancy
	21	18	f	lat	160	4	yes	2	
	22	27	m	med. + lat	80	4	yes	1	
	23	20	m	med	60	2	**no**	3	regenerate insufficiancy
	24	46	m	med	220	5	yes	6	
	25	51	m	med	91	7	**no**	6	ventral impingement

*No.* number, *y* years, *m.* male, *f.* female, *med.* medial, *lat.* lateral

## Discussion

The most important finding of this study is that treatment of osteochondral lesions of the talus via minced cartilage can lead to excellent radiological outcomes in the majority of patients (80%). The primary outcome AOSS reached excellent (3.2 ± 2.5) values in all patients within a one-year follow up via MRI. Complication-rate, the secondary outcome, was dependent on the fixation method. It remains low, but only if fibrin is used as a top layer fixation method. This was demonstrated by the high rate of revision surgery in the MCAC patients (56%), whereas none of the patients with a complication had received fibrin-fixation intraoperatively. This difference in secondary outcome between treatment modalities was statistically significant in this study.

The primary goal of the reconstruction of osteochondral defects at the talus is the reformation of hyaline cartilage at the site of defect. With the use of minced cartilage, there is no need to cultivate the articular chondrocytes in vitro after harvesting. Cells do not need to be expanded before implantation, which is not just a time consuming and expensive procedure. While immature chondrocytes seem to produce good extracellular matrix in animal studies [18], adult chondrocytes cells have been demonstrated to dedifferentiate during expansion. The result are cell populations that do not seem to be able to express and deposition matrix molecules to form a stable and well-functioning cartilage [19].

A well-known problem of articular cartilage is its poor intrinsic capacity for repair, especially in lesions that do not penetrate the subchondral bone [20]. Therefore, a concomitant treatment of the subchondral bone should be performed in all cases. This can be achieved by debridement and/or implanting drill holes, as well as implanting autologous cancellous bone before implanting the minced cartilage fragments. Histologic studies have observed apoptosis in defect edges emigrating from the direction of cartilage lesion boarders [21]. There has been shown to be less apoptosis and disruption of the matrix in cartilage lesion edges however, when cut with a sharp knife. In addition, signs of collagen and sulphated glycosaminoglycan synthesis immediately adjacent to the lesion edge were observed [22]. Outgrowth of fibrin and collagen hydrogels has also been observed in cartilage fragments after the use of a mincing device in recent scientific efforts [19]. This supports the use of a fresh scalpel to harvest the cartilage as well as for the cutting procedure or the additional use of a mincing device with sharp blades.

In this study, after reconstruction of the required bone stock via autologous cancellous bone from the proximal tibia, distal tibia or iliac crest, cartilage was harvested from the defect site. It was then cut into smaller fragments and reimplanted with the preferred refixation method. Prerequisite for a successful graft integration is a sufficient bone stock and a good blood circulation as well as a straight ankle joint axis. The bone stock as a base layer for the cartilage graft can be build up with autologous cancellous bone for example from the tibia or iliac crest. If the defect cannot be reached adequately, an additional osteotomy should be considered.

**Fig. 4:**
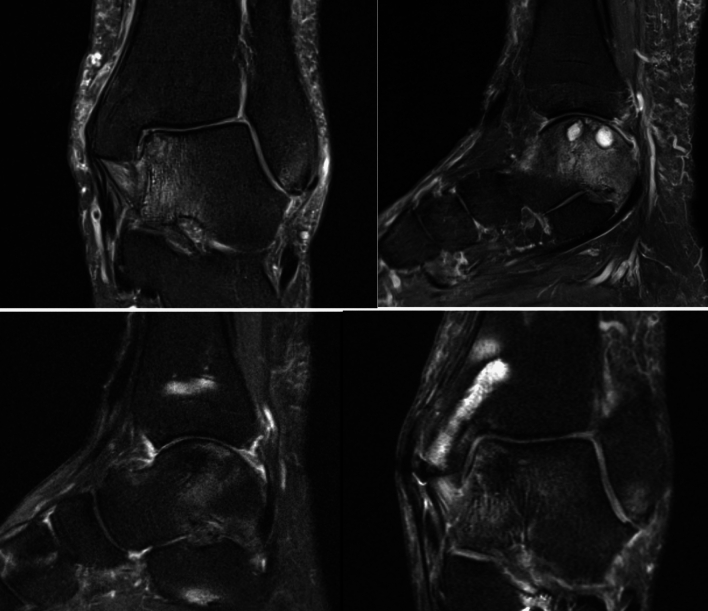
Coronary and sagittal slices of t2 sequenced MRI preoperatively and postoperatively after treatment of an osteochondral defect with subchondral cysts in the medial talus with autologous cancellous bone from the proximal tibia and minced cartilage along with fibrin fixation after osteotomy of the distal tibia

An important radiological indicator for the success of the procedure is the progressive coverage of bone stock with cartilage and a low water signal in t2-sequences in MR imaging. This relates with cartilage cell outgrowth, proliferation, and differentiation (Fig. 4). Indirect radiological parameters are the amount of bone marrow edema, joint effusion and synovialitis. Direct and indirect radiological parameters are used to evaluate AOSS scores.

So far there are no studies reporting AOSS scores after cartilage repair techniques in the ankle. In 2015 Schmal et. al. presented a study of 40 adult patients with osteochondrosis dissecans with a mean AOSS of 10.2 ± 3.4 [16]. A different study could demonstrate that a good clinical follow up score is associated with a good score in the AOSS [23]. The AOSS values (3.2 ± 2.5) within one year after the minced cartilage procedure from this study can therefore be considered excellent. Overall, there was no relevant difference outcome in the AOSS or the surgical revision rate for different locations of the osteochondral defects noted in this study. Radiological consolidation parameters showed successful healing of the graft across both genders and all age groups in form of bony graft integration as well as cartilage coverage, which is in line with recent studies evaluating the procedure in the knee joint [3].

Interestingly, data from this study revealed an accumulated occurrence of complications in the form of insufficient graft integration, osteophytes, and consecutive revision surgery in the MCAC group. Even more interestingly, this occurred in all patients that did not receive fibrin fixation as an additional measurement. This led to an elevated revision rate in this group, which was independent of defect size, -depth and -location. In contrast to that, there were no complications documented for MCF (and MCMF) patients. The authors could demonstrate excellent ingrowth of the cells independent of the exact method or defect size if a fibrin clot was formed as last step fixation. There was no graft dislocation detectable via MRI after MCF or MCMF within 12 months after surgery. The use of fibrin alone therefore seems to result in sufficient fixation of the cancellous bone and minced cartilage fragments even without the additional use of a membrane or PRP. This has the advantage that chondrocytes do not need to digest the acellular membrane before forming a stable construct. The necessity for the addition of a membrane or PRP should therefore be discussed. This is in line with recent studies, that questioned the benefit of additional use of PRP on the outcomes after autologous chondrocyte implantation in a rabbit model [24]. Minced cartilage with fibrin fixation only as a one-step procedure, however, is cost-efficient and produces excellent healing rates within a period of 12 months in patients with an osteochondral defect of the talus, independent of the defect size. The use of fibrin alone seems to result in sufficient fixation of the cancellous bone and minced cartilage fragments even without the additional use of a membrane. In contrast to two-step procedures, a second hospitalization is obsolete, and rehabilitation protocols can be initiated right away.

### Limitations

This study has several limitations, starting with its retrospective design and the fact that it is a report of a case series without any comparison between treatment modalities. It presents short-term radiological outcomes of the first series of patients undergoing minced cartilage in the ankle and therefore lacks a comparative group of patients treated with alternative procedures for osteochondral defects. The decision to carry out a specific procedure was the individual choice of the surgeon at the time of surgery. No mid-term or long-term outcomes were evaluated. No clinical outcome other than the surgical revision rate was evaluated. Correlation analysis with patient reported outcome measures (PROMS) will be of special importance to confirm AOSS values derived from MRI in the ankle after the minced cartilage procedure in the future. Because of the small sample size, statistical subgroup analyses were not possible. A consecutive number of cases with good radiological consolidation rates and low revision rate over the documented period were presented and scientifically evaluated however, which has not been done before. Further larger prospective studies with mid-term and long-term as well as clinical outcomes are necessary to finally classify this relatively new method in the ankle joint in its role in modern cartilage surgery of the foot and ankle.

## Conclusions

Autologous minced cartilage implantation seems to be sufficient as a viable, one-step treatment for osteochondral lesions of the talus. It leads to low complication rates and excellent AOSS values within a 12-month period whereat the application of fibrin as the last fixation layer seems to be advisory, which demands further investigation.

## Supplementary Information

Below is the link to the electronic supplementary material.

Supplementary file1 (DOCX 15 KB)

## Data Availability

No datasets were generated or analysed during the current study.

## Abbreviations

%: Percent
AOSS: Ankle-osteoarthritis-scoring-system
CI: Confidence interval
f.: Female
Fig.: Figure
lat.: Lateral
m.: Male
MCAC: Minced cartilage with autocart-procedure
MCF: Minced cartilage with fibrin
MCMF: Minced Cartilage with membrane and fibrin
med.: Medial
mm: Milimeter
MR: Magnet-resonance
MRI: Magnet-resonance-tomography
n: Number
PROM: Patient reportet outcome measures
PRP: Platelet rich plasma
Q–Q: Quantile–quantile
y.: Years
