# Supplementary material for: Autologous minced cartilage implantation in osteochondral lesions of the talus—does fibrin make the difference?
Source: Arch Orthop Trauma Surg. 2025 Jan 25;145(1):144. doi: 10.1007/s00402-025-05762-7 (PMC11761507; doi:10.1007/s00402-025-05762-7)
Supplement: Supplementary file 1 — Supplementary file1 (DOCX 15 KB) [file 402_2025_5762_MOESM1_ESM.docx]

**Autologous Minced Cartilage Implantation in Osteochondral Lesions of the Talus – Does Fibrin Make the Difference?**

J. Kühle^1^, F.C. Wagner^1^, S. Beck^1^, L. Klein^1^, L. Bode^1^, K. Izadpanah^1^, H. Schmal^1,2^, N. Mühlenfeld^1^

1. *Department of Orthopedics and Trauma Surgery, Medical Centre - Albert-Ludwigs-University of Freiburg, Faculty of Medicine, Albert-Ludwigs-University of Freiburg, Germany*
2. *University Hospital Odense, Dep. Of Orthopedic Surgery, Sdr. Boulevard 29, 5000 Odense C, Denmark*

Corresponding author

PD Dr. med. Nils Mühlenfeld MHBA

Department of Orthopedic Surgery and Traumatology

Albert Ludwigs University Freiburg

Hugstetter Straße 55

D-79106 Freiburg

Tel.: +49-270 24010

E-Mail: nils.muehlenfeld@uniklinik-freiburg.de

ORCID: 0000-0002-4226-5934
